# Effect of standardized nursing language continuing education programme on nurses' documentation of care at University College Hospital, Ibadan

**DOI:** 10.1002/nop2.108

**Published:** 2017-11-27

**Authors:** Iyanuoluwa O. Adubi, Adenike A. Olaogun, Prisca O. Adejumo

**Affiliations:** ^1^ Clinical Nursing Department University College Hospital Ibadan Nigeria; ^2^ Department of Nursing Science Obafemi Awolowo University Ile‐Ife Nigeria; ^3^ Department of Nursing University of Ibadan Ibadan Nigeria

**Keywords:** education, language, nurses, nursing, terminology

## Abstract

**Aim:**

The study assessed the documentation of nursing care before, during and after the Standardized Nursing Language Continuing Education Programme (SNLCEP). It evaluates the differences in documentation of nursing care in different nursing specialty areas and assessed the influence of work experience on the quality of documentation of nursing care with a view to provide information on documentation of nursing care. The instrument used was an adapted scoring guide for nursing diagnosis, nursing intervention and nursing outcome (Q‐DIO).

**Design:**

Retrospective record reviews design was used.

**Methods:**

A total of 270 nursing process booklets formed the sample size. From each ward, 90 booklets were selected in this order: 30 booklets before the SNLCEP, 30 booklets during SNLCEP and 30 booklets after SNLCEP.

**Results:**

Overall, the study concluded that the SNLCEP had a significant effect on the quality of documentation of nursing care using Standardized Nursing Languages.

## 1 INTRODUCTION

In Nigeria, nursing process and the use of NANDA‐I nursing diagnoses have been incorporated into nursing education since the 1980s, but the concepts of Nursing Outcome Classification (NOC) and Nursing Intervention Classification (NIC) are relatively new and nurses knew little about Standardized Nursing Languages (SNLs). These concepts were introduced into the country through an international workshop hosted by the NANDA‐I African Network‐Nigeria Chapter, NANDA‐I and the Center for Nursing Classification and Clinical Effectiveness of the College of Nursing, University of Iowa, in 2010. This study is a by‐product of the workshop held at University College Hospital, Ibadan.

### 1.1 Background of the study

Keenan (1999) observed that throughout history nurses have documented nursing care using individual and unit‐specific methods; consequently, there is a wide range of terminology to describe the same care. Nurses lacked a standardized language to communicate their practice until the North American Nursing Diagnosis Classification (NANDA) was introduced in 1973 Rutherford (2008). However, the development of SNLs began in the early 1970s, and has been and continues to be an ongoing process (Lunney, 2009). Nursing diagnoses, development began in 1973 when a group of nurse leaders assembled to identify a system of labels to describe nursing practice (Johnson et al., 2006).

According to NANDA‐I (2012‐2014), nursing diagnosis is a clinical judgement that provides the basis for selection of nursing interventions to achieve outcomes for which the nurse are accountable. The Nursing Intervention Classification (NIC), a classification system specific for nursing interventions, was first published in 1992 and included 336 interventions (Iowa Intervention Project, 2008). An intervention is defined as “any treatment, based upon clinical judgment and knowledge that a nurse performs to enhance patient/client outcomes” (Bulechek, Butcher, Dochterman, & Wagner, 2013. Pg 6), while an individual nurse will have expertise in only a limited number of interventions reflecting on her or his specialty, the entire classification captures the expertise of all nurses.

The Nursing Outcome Classification (NOC) is a nursing classification system with specific labels used to describe outcomes for a client or group of clients. The development of the NOC was first published in 1997 (Iowa Outcomes Project, 2008). An outcome is a measurable individual, family or community, state, behaviour or perception that is measured along a continuum and is responsive to nursing interventions (Moorhead, Johnson, Maas, & Swanson, 2013). The three components which are NANDA, NIC and NOC make up Standardized Nursing Language (SNL). Hence, SNL is “a structured vocabulary that provides nurses with a common means of communication to describe care” (Beyea, 1999). Hardiker, Hoy, and Casey (2000), argue that terminological standards in nursing (example NOC, NIC and NANDA) are one of the major tools used by the nursing profession to establish its autonomy as well, making nursing visible, given that these standards presents the range of nursing tasks that are over shadow by the works of physicians. The important thing is that these standards are necessary to achieve a system that supports nursing work in a multidisciplinary setting and representation of its outcome in health information systems (Hardiker et al., 2000).

Bulechek, Butcher, & Dochterman, 2008 reported that the use of this SNL in nursing documentation can result in better continuity of care by improving communication among nurses (other healthcare providers) and capture more nursing activities as evidence to determine nursing costs, provide standards for improving the quality of nursing care and allow data collection which helps in evaluating the patient outcomes of nursing care.

Falk and Bjorvell (2012) stated in a study conducted that, by assessing part of the nursing process it was relatively well documented in the patient record, although the analysis resulting in a nursing diagnosis, in Sweden written in free text format, generating a useful care plan is virtually non‐existent. Meanwhile, nursing process and nursing diagnosis have a prominent position in the curricula of most institutions of higher education for nursing; it appears difficult to apply in clinical practice (Carpenito‐Moyet, 2010).

According to Muller‐Staub, Lavin, Needham, & van Achterberg, 2006, the use of SNLs in documenting care has improved the image of the nursing profession in developed nations. Several studies have been done globally, which are hospital‐based care on documentation of care using SNLs (Bakken, 2007; Falk & Bjorvell, 2012; Muller‐Staub, Needham, Odenbreit, Lavin, & van Achterberg, 2007; & Olaogun, Oginni, Oyedeji, Nnahiwe, & Olatubi, 2011).

Koczmara, Jelincic, and Dueck (2005) did a study which examined the accuracy of nursing documentation and inaccuracy of nursing documentation, the conclusion stated that nurses misinterpret thus putting patients in unsafe situations. However, a recent report by Odutayo, Olaogun, Oluwatosin, & Ogunfowokan (2013) stated that public health nurses in Nigeria at a posttest were able to identify actual nursing diagnoses with the signs and symptoms and aetiologies, while in the risk diagnoses they identified the nursing diagnoses with the risk related factors. They also identified NIC activities specific to solving client problems and linked indicators of NOC, which were related to the identified diagnoses and interventions. Bakken (2007) stated that in most developing countries especially sub‐Saharan Africa nursing documentation is not at its best; she also noted that large numbers of under qualified nursing staff, lack of incentives such as reimbursement, accreditation among others that are attached to efficient documentation barely exist in developing countries. However, documentation is core to effective use of the nursing process for quality nursing care, but nurses have been noted to be poor in the extent which they document the care they give, especially related to use of appropriate nursing terminologies (Ammenwerth, Mansmann, Iller, & Eichstädter, 2003). Ammenwerth et al. (2003) stated that one of the greatest shortcomings of nursing has been its failure to clearly define and specifically delineate its functions and unique contributions to patient care using a SNL and this is currently the situation among clinical nurses. It is also observed that nursing is at a crossroad and this is no more evident than in discussions around the use of the languages, nursing diagnoses, interventions and outcomes in documentation of client care (Olaogun et al., 2011).

Unfortunately, in Nigeria, many nurses oppose the use of SNLs, stating “It's just another label” while they see no problem with the use of medical labels such as malaria or hip fracture. They feel the use of labels that articulate the unique patient problem from a nurse's perspective is redundant, vague and trivial (Olaogun et al., 2011). Moreover, Thoroddssen and Ehnfors (2007) introduced SNL, such as NANDA and they stated that this has improved the quality of nursing documentation in the patient record.

Odutayo et al. (2013) stated that, without the use of SNLs in documentation, the clinical reasoning and decision‐making processes of nursing are obscured. They also said that nurses' risk functioning in a way that is more task driven with little disciplinary focus and makes nursing become hidden in a system that is dominated by medical care, while other healthcare disciplines are focused on advancing their science and the care they deliver, nursing responds by becoming less visible and assuming roles that make care contributions ambiguous and indistinct (Odutayo et al., 2013).

Additionally, despite the training undergone by nursing staff of the University College Hospital, they often expressed difficulties about documenting nursing care according to the standardized terminologies in the nursing process booklets (NPBs). Hence, the researchers considered it worthwhile to measure the effect of standardized nursing language continuing education programme (SNLCEP) on documentation of nursing care at University College Hospital, Ibadan.

### 1.2 Design

The study employed was a retrospective record review design.

## 2 METHODS

The study was conducted in three wards at University College Hospital (UCH), southwestern Nigeria. UCH is strategically located in Ibadan, the largest city in West Africa, which is the seat of the premier University in Nigeria. There are 14 units in the clinical department of nursing, University College Hospital, Ibadan; using purposive sampling technique, three wards were selected from the 14 units which include medical unit, surgical unit and psychiatric unit. Therefore, 270 NPBs were spread over the units; 90 NPBs were selected purposefully. The documented booklets were kept at the nurses' station.

The instrument used for data collection was the modified Muller‐Staub et al. (2007) measurement instrument. The instrument can be seen in the appendix. The measurement instrument was modified into two sections.

Section A: Demographic characteristics developed by the researcher to elicit information about the professional characteristics of nurses who documented the selected nursing process booklets. This included the work experience, wards, educational qualification, specialization and workshop.

Section B: Items of the measurement instrument were adapted from Muller‐Staub et al. (2007).

It measures the documentation of nursing diagnosis, nursing interventions and outcomes. The “Nursing diagnoses as process” has 11 items measured on a 3‐point Likert‐type scale (2–0) with maximum score of 22, while “Nursing diagnoses as a product” has eight items measured on a 5‐point Likert‐type scale (4–0) with maximum score of 32. Furthermore, “Nursing intervention” has three items measured on a 5‐point Likert‐type scale (4–0) with maximum score of 12 and “Nursing outcomes” has seven items measured on a 5‐point Likert scale (4–0) with maximum score of 28. The total item is 29 and total score is 94. The scoring was rated Low, Medium and High scores: Low‐quality documentation (scores from 22–46), Medium‐quality documentation (scores from 47–70) and High‐quality documentation (scores from 71–94).

The content of SNLCEP provided was through an educational package that was developed by the authors from literature review and learning modules that were produced and used during the 2010 international workshop on NANDA‐I diagnostic classification, NOC and NIC (Brokel & Herdman, 2010a, 2010b, 2010c; Butcher, 2010; Moorhead, 2010). This package is made up of four learning modules.

Module 1—Historical development of Standardized Nursing Languages.

Module 2—Introduction to the concepts of NANDA‐I nursing diagnoses, NOC and NIC.

Module 3—Linkages between the Nursing Diagnoses, Nursing Outcome Classifications and Nursing Intervention Classifications (NNN).

Module 4—Documentation of care using NNN in documentation.

The nurses underwent SNLCEP. Nurses from medical ward, surgical ward and psychiatric ward had 2 months of teaching in each ward and from the 7th month the nurses at the three units came together and they were educated again; Two of the facilitators involved in the training of these nurses are PhD holders and have undergone postdoctoral studies on the NNN at the College of Nursing, University of Iowa, USA. Hence, the SNLCEP covered a period of 7 months (January 2012–July 2012) and the hours of teaching were 2 hr 30 min, and this same set of nurses resume back to their wards to carry out their nursing activities for the day and they are expected to use the training undergone on their wards. Furthermore, the same nurses' documentation was assessed after SNLCEP within 6 months (August 2012–January 2013).

The study was later conducted in four stages.

Stage 1: The wards to be used were identified, i.e. medical, surgical and psychiatric. A total of 270 NPBs were spread over three wards. Ninety NPBs were purposively selected from three wards (30 each). Numbers of NPBs picked before SNLCEP was 90, during SNLCEP were 90 and after SNCLEP were 90. Duration of a week was used for the documentation. Meanwhile, the NPBs of nurses who attended the training was sought for on the wards and assessed, during and after SNLCEP. However, the same set of nurses who documented in NPBs prior SNLCEP was also retrieved on the wards and same assessed.

Stage 2: All NBPs for the period prior to SNLCEP (July 2011–August 2011) were assessed using the Q‐DIO measurement instrument. All raw data were entered into the computer.

Stage 3: All NBPs covering the period during (January 2012–July 2012) were assessed using the Q‐DIO. All raw data were entered into the computer.

Stage 4: All NBPs covering the period during (August 2012–January 2013) were assessed using the Q‐DIO. All raw data were entered into the computer.

Data were analysed using the Statistical Package for Social Sciences (SPSS) version 16.0 for both descriptive and inferential statistics.

### 2.1 Ethics

Ethical clearance was obtained from the Institute for Advanced Medical Research and Training, College of Medicine, University of Ibadan, Nigeria. Approval number NHREC/05/01/2008a. Permission was also taken from the ward coordinators and chief nursing officers of the selected wards. However, informed consent was taken from the nurses involved in documentation.

## 3 RESULTS

As shown in Table 1, 270 nurses attended the SNLCEP, and this same set of nurses documented in the NPBs. The number of NPBs documented in on each ward was 90 each. However, none of the nurses who documented in NPBs had a postgraduate degree in nursing. Their highest nursing education is Diploma in Nursing. The nurses' with 1–5 years of experience documented more in the NPBs.

**Table 1:** Professional characteristics of nurses who documented in the nursing process booklets

	*N*	Percentage (%)
Work experience (in years)		
1–5	179	66.3
6–10	76	28.2
10 and above	15	5.6
Total	270	100
NPBs documented in each Units		
Medical	90	33.3
Surgical	90	33.3
Psychiatric	90	33.3
Total	270	100
Nurses who attended SNLCEP	270	100
Total	270	100
Educational qualification		
Diploma	194	71.9
Bachelor in nursing science	76	28.2
Postgraduate (in nursing)	0	0
Total	270	100

As shown in Table 2, the mean score of the documentation after the SNLCEP was 72.28 (*SD* 14.74). This showed that SNLCEP had an effect on nurses documentation of care. As shown in Table 3, the surgical ward had a mean of 71 (*SD* 14.97); hence the SNLCEP improved quality of documentation on the surgical ward, followed by psychiatric ward and medical ward. Table 4 shows that there was significant difference in documentation of nursing care among the ward. As shown in Table 5, nurses with 1–5 years of experience had a mean of 65.25 (*SD* 16.26). However, the nurses with 1–5 years had an improved quality of documentation of nursing care. As shown in the Table 6, the chi‐square test result showed that there was no significant difference, with x_2_ = 2.57, *df* = 4 and p value greater than 0.05.

**Table 2:** Documentation of nurses before, during and after standardized nursing language continuing education programme

Periods	Mean	*N*	Standard Deviation
Before	60.08	90	10.94
During	59.53	90	18.30
After	72.28	90	14.74
Total	63.96	270	16.03

**Table 3:** The result of the mean and standard deviation of quality of documentation of nursing care in the units (medical, surgical and psychiatric) by nurses who attended SNLCEP

Periods	Units	Mean	*N*	Standard Deviation
Before	Medical	49.7667	30	7.80664
	Surgery	66.7333	30	10.24841
	Psychiatric	63.7333	30	5.60131
	Total	60.0778	90	10.93518
During	Medical	43.7667	30	16.08530
	Surgery	66.9667	30	15.94707
	Psychiatric	67.8667	30	11.33117
	Total	59.5333	90	18.29852
After	Medical	66.9000	30	13.99347
	Surgery	80.6333	30	14.04054
	Psychiatric	69.3000	30	12.74187
	Total	72.2778	90	14.73819
Total	Medical	53.4778	90	16.28277
	Surgery	71.4444	90	14.96746
	Psychiatric	66.9667	90	10.51639
	Total	63.9630	270	16.02990

**Table 4:** ANOVA table showing results of quality of documentation of nursing care on the units (medical surgical and psychiatric)

			Sum of squares	*df*	Mean square	*F*	Sig.
Quality of documentation of nursing care on the units.	Between Groups	(Combined)	9346.719	2	4673.359	20.875	.000
	Within Groups		59774.911	267	223.876		
	Total		69121.630	269			

**Table 5:** Showing results of quality of documentation of nursing care with years of experience of nurses (1 to above 10) years

Periods	Years of work experience	Mean	*N*	Standard Deviation
Before	(1–5)years	59.4727	55	11.12697
	(6–10)years	60.3667	30	10.64630
	Above 10 years	65.0000	5	11.46734
	Total	60.0778	90	10.93518
During	(1–5)years	62.7581	62	17.83594
	(6–10)years	52.5000	26	17.51628
	Above 10 years	51.0000	2	25.45584
	Total	59.5333	90	18.29852
After	(1–5)years	72.8710	62	15.76309
	(6–10)years	72.1000	20	8.62615
	Above 10 years	68.1250	8	19.27572
	Total	72.2778	90	14.73819
Total	(1–5)years	65.2514	179	16.26150
	(6–10)years	60.7632	76	14.93173
	Above 10 yrs	64.8000	15	17.41182
	Total	63.9630	270	16.02990

**Table 6:** Showing result of work experience on quality of documentation of care

	Value	*df*	Sig.
Work experience on quality of documentation	2.567	4	.633

## 4 DISCUSSION

In Nigeria, nursing process and the use of NANDA‐I nursing diagnoses have been incorporated into nursing education since the 1980s, but the concepts of the NOC and NIC are relatively new and nurses knew little about SNL. These concepts were introduced into the country through an international workshop hosted by the NANDA‐I African Network‐Nigeria Chapter, NANDA‐I and the Center for Nursing Classification and Clinical Effectiveness of the College of Nursing, University of Iowa, in 2010. This study is a by‐product of the workshop held at University College Hospital, Ibadan. As reported by Muller‐Staub et al. (2007), von Krogh and Nåden (2008) and Muller‐Staub et al. (2009) in similar research conducted on the use and documentation of SNLs, with the introduction of adequate education and resources, nursing documentation increases with use of SNLs. Muller‐Staub et al. (2007) and Criminiello, Terjesen, & Lunney (2009) discovered that before the implementation of NNN, nursing problems were formulated in freestyle without the use of standardized classification. But after SNLCEP, data showed significant improvement in documentation. Abreu (2006) and Hughes (2006) also reported the use of NNN in documenting the care of orthopaedic and spinal cord injury patients in Brazil and Ireland. NNN linkages are, therefore, an important step in the organization of nursing information. Furthermore, findings support the study of Odutayo et al. *(* 2013) which revealed that there was an increase in the documentation of care by public health nurses working in primary health care centres in Ogun State after the introduction of an educational programme on SNLs. This is also in line with the study carried out by Muller‐Staub et al., 2007 in a Swiss hospital among nurses who received educational interventions, and the result showed significant enhancement in the quality of documentation of nursing care among nurses. This is consistent to Adeyemo and Olaogun (2013) who reported that the more nurses are knowledgeable, the more their use of nursing process.

The findings from the study also provided insight into the quality of documentation of nursing care in medical, surgical and psychiatric wards among participants after SNLCEP, which were significant. This is consistent with the study conducted in Ahmadu Bello University Teaching Hospital that showed that there was significant difference in quality of documentation of nursing care in the level of implementation of NPBs across units such as medical, surgical, obstetrics and gynaecology, paediatrics and special units after educational package (Garba et al.*,* 2011 as cited by Edet, Mgbekem, & Edet, 2012). It was reflected in the study that the quality of documentation of nursing care among nurses with various work experiences was not significant. This is at variance with report of the study conducted by Sani and Sani (2013). They found that there was a significant difference between knowledge of SNL and work experience among nurses. One can infer from this study that no matter how experienced the nurses are, it does not improve nor depreciate their knowledge of SNL and documentation of nursing care. Their knowledge does not improve with experience. This might be due to the fact that nurses are not able to apply the theoretical knowledge acquired in school. Also, it might be lack of desire or unwilling attitude put up by some nurses to upgrade their knowledge after qualified to practice nursing. Akintaju (2012) discovered that the more their years of experience, the better their knowledge of the patient condition and documentation of care. After SNLCEP, the nurses as reflected in their documentation were able to report actual nursing diagnoses with the signs and symptoms and aetiologies, while in the risk diagnoses they identified the nursing diagnoses with the risk factors. Also, they identified NIC activities specific to solving client problems and linked indicators of NOC, which were related to the identified diagnoses and interventions.

The American Nurses Association (2007) stated that nurses would acquire adequate knowledge when trained consistently through seminars/workshops, in‐service training and higher education, and able to identify nursing diagnoses and related factors. Similarly, Rogers (2005) suggests that individuals and groups make decisions to adopt new technology, ideas and practices when they obtain knowledge and develop their attitude. Therefore, practitioners, including nurses, would accept and implement new ideas and practices, such as the use of SNLs, if they are informed, educated and have a better understanding of the relevant concepts. Paans, Muller‐Stuab, and Nieweg (2013) stated that there is the need to ensure regular in‐service training for nurses to improve their knowledge of SNLs. Also, training nurses to improve knowledge, skills and documentation practices has been a widely used strategy to improve documentation quality as revealed in study conducted by Jefferies, Johnson, & Griffiths, 2012.

This is the first reported empirical study using NNN in client care in a Nigeria hospital. It is anticipated that future research will be conducted in non‐hospital settings to test the effect of the NANDA‐I diagnoses, NIC, NOC (NNN), on the quality of nursing care and its documentation.

## 5 CONCLUSION

The findings of this study accentuate the fact that predominantly in constant use is the NANDA, while the use of activities in the NIC and NOC was in existence among the nurses. This study has demonstrated that there was a medium quality of documentation on medical ward and surgical ward, while psychiatric ward had high‐quality documentation as regard SNLs. Also, there was no significant difference in the quality of documentation of nursing care and nurses work experience.

However, significant efforts have been made to unify SNLs through the taxonomy of NNN. Furthermore, this study has demonstrated that the education of nurses on the nursing process and the implementation of SNLs through the SNLCEPs is a viable way to improve nurse skills in the documentation of care.

The NPBs were difficult to retrieve due to poor record keeping by nurses. The literatures reviewed on studies done on SNLs were based on the available local studies. There is a need for more research in Nigeria and other African nations on how SNLs can be adapted to client care and the effect of SNLs on the quality of nursing care.

## CONFLICT OF INTEREST

No conflict of interest has been declared by the authors.

## APPENDIX 1

### Section A The demographic data of nurses

1. Work experience (in years) (a) 1–5 (b) 6‐10 (c) 10 and above
2. Units (a) Medical (b) Surgical (c) Psychiatric
3. Educational qualification (a) Diploma (b) Bachelor in nursing science (c) postgraduate in nursing.

### Section B Measurement instrument q‐dio

**Table:**

Dimensions/Items	3‐point scale				
Nursing diagnoses as processInformation is documented about:	2		1	0	
1. Actual situation, leading to the hospitalization					
2. Anxiety and worries related to hospitalization, expectations and desires about hospitalisation					
3. Social situation and living environment/circumstances					
4. Coping in the actual situation/with the illness					
5. Beliefs and attitudes about life (related to the hospitalization)					
6. Information of the patient and relatives/significant others about die situation					
7. Intimacy, being female/male					
8. Hobbies, activities for leisure					
9. Significant others (contact persons)					
10. Activities of daily living					
11. Relevant nursing priorities according to the assessment					
11 Items, maximum score = 22, mean = 2					
Nursing diagnoses as product	5‐point scale				
	4	3	*2*	1	1
12. Nursing diagnosis label is formulated					
13. Nursing diagnosis labels is formulated according to NANDA and is numbered					
14. The aetiology (E) is documented					
15. The aetiology (E) is correct, related/corresponding to the nursing diagnosis (P)					
16. Signs and symptoms are formulated					
17. Signs and symptoms (S) arc correctly related to the nursing diagnosis (P)					
18. The nursing goal relates/corresponds to the nursing diagnosis					
19. The nursing goal is achievable through nursing interventions					
8 Items, maximum score = 32, mean = 4					
Nursing interventions	4	3	*2*	1	0
20. Concrete. clearly named nursing interventions ‐ according to Doenges/Moorhouse ‐ are planned (what will be done. how. how often, who does it)					
21. The nursing interventions effect the aetiology of the nursing diagnosis					
22. Nursing interventions carried out are documented (what was done. how. how often, who did it)					
3 Items, maximum score = 12, mean = 4					

Q‐DIO to be used by authors permission. Citation references:Muller‐Staub, M., Lunney, M., Odenbreit, M., Needham, 1., Lavin, M. A., & van Achterberg, T. (2009). Development of an instrument to measure the quality of documented nursing diagnoses, interventions and outcomes: the Q‐DIO. Journal of Clinical Nursing, 18(7), 1027‐1037. Muller‐Staub, M., Lunney, M., Lavin, M. A, Needham, I., Odenbreit, M., & van Achterberg, T. (2008). Testing the Q‐DIO as an instrument to measure the documented quality of nursing diagnoses, interventions, and outcomes. International Journal of Nursing Terminologies and Classifications, 19(1), 20‐27.
